# Evaluation of a Wearable in-Ear Sensor for Temperature and Heart Rate Monitoring: A Pilot Study

**DOI:** 10.1007/s10916-022-01872-6

**Published:** 2022-11-04

**Authors:** David Benjamin Ellebrecht, Damian Gola, Mark Kaschwich

**Affiliations:** 1 Department of Surgery, University Medical Center Schleswig-Holstein, Campus Luebeck, Ratzeburger Allee 160, 23538 Luebeck, Germany; 2 Institute of Medical Biometry and Statistics, University of Lübeck, Ratzeburger Allee 160, 23562 Luebeck, Germany; 3 Department of Thoracic Surgery, LungenClinic Großhansdorf, Woehrendamm 80, 22927 Grosshansdorf, Germany; 4 Department of Vascular Medicine, University Heart & Vascular Centre Hamburg, Martinistraße 52, 20246 Hamburg, Germany

**Keywords:** Body core temperature, Heart rate, Ear-sensor, Thermometer, Postoperative monitoring, Wearable sensors

## Abstract

In the context of the COVID-19 pandemic, wearable sensors are important for early detection of critical illness especially in COVID-19 outpatients. We sought to determine in this pilot study whether a wearable in-ear sensor for continuous body temperature and heart rate monitoring (Cosinuss company, Munich) is sufficiently accurate for body temperature and heart rate monitoring. Comparing with several anesthesiologic standard of care monitoring devices (urinary bladder and zero-heat flux thermometer and ECG), we evaluated the in-ear sensor during non-cardiac surgery (German Clinical Trials Register Reg.-No: DRKS00012848). Limits of Agreement (LoA) based on Bland–Altman analysis were used to study the agreement between the in-ear sensor and the reference methods. The estimated LoA of the Cosinuss One and bladder temperature monitoring were [-0.79, 0.49] °C (95% confidence intervals [-1.03, -0.65] (lower LoA) and [0.35, 0.73] (upper LoA)), and [-0.78, 0.34] °C (95% confidence intervals [-1.18, -0.59] (lower LoA) and [0.16, 0.74] (upper LoA)) of the Cosinuss One and zero-heat flux temperature monitoring. 89% and 79% of Cosinuss One temperature monitoring were within ± 0.5 °C limit of bladder and zero-heat flux monitoring, respectively. The estimated LoA of Cosinuss One and ECG heart rate monitoring were [-4.81, 4.27] BPM (95% confidence intervals [-5.09, -4.56] (lower LoA) and [4.01, 4.54] (upper LoA)). The proportion of detection differences within ± 2BPM was 84%. Body temperature and heart rate were reliably measured by the wearable in-ear sensor.

**Supplementary Information:**

The online version contains supplementary material available at 10.1007/s10916-022-01872-6.

## Introduction

Facing the daunting coronavirus 2019 (COVID-19) pandemic in the last two years, with limited ICU beds, resources and health care personnel, it is essential to detect critical illness in COVID-19 patients before they begin to experience shortness of breath to prevent the pneumonia from progressing to a dangerous level [1]. Especially, there is a need of continuous monitoring of hospitalized or outpatient COVID-19 patients. Thus, defined attributes of an ideal monitoring should be non-invasiveness, accuracy during large changes in physiological conditions, independence of operator and technique, simplicity of use, and provide continuous monitoring [2].

Wearable sensors have been widely used for fitness tracking as well as daily life and may be used to continuously monitoring for the detection if worsening symptoms. Detecting patients with threatening respiratory deterioration, wearable sensors could bridge the gap between home isolation and normal ward, respectively, and the need for ICU. On the other hand, wearable sensors have the potential for monitoring healthy individuals who had exposure [3]. In this context, body temperature and heart rate play a crucial role for monitor early warnings signs. Body temperature rises during a viral infection due to immune activation. Therefore, body temperature monitoring has been widely accepted for detection of early signs of Covid-19 infection, and several wearable devices are available for monitoring of individuals who had the risk of exposure [4]. Due to a viral infection an increase of heart rate and change in pulse waveforms indicate physiological stress. Several studies showed the feasibility of wearable devices for continuous heart rate monitoring and detection of cardiac events [5, 6]. For instance, using a Fitbit or a Huami sensor, two studies showed the prediction of influenza-like illness by analyzing the resting heart rate [7, 8]. Studies have also indicated that COVID-19 infection are associated with cardiac events like arrhythmias, heart failure and myocarditis, for instance [9].

However, different devices are required for continuous monitoring vital signs and the challenge with these solutions is that the gadgets they use can only measure or track one or two symptoms of COVID-19. Additionally, there is a lack of data evaluating the available wearables devices with the standard of care monitoring devices. Passler et al. demanded studies with respect to measurement accuracy and precision [10]. Continuous ECG is the general accepted standard of care of intraoperative and ICU heart rate monitoring. Continuous body temperature measurement turns out to be more complicated, because there is an ongoing discussion of the most accurate body temperature monitoring site [11]. Although there are different monitoring sites the implantation of continuous non-invasive body temperature monitoring is difficult [12–14]. In general, there is a lack of data proving sufficient measurement accuracy of the wearable sensors compared to the standard of care monitoring devices.

Therefore, the purpose of this observational study is the evaluation of the new-developed in-ear sensor for continuous body temperature and heart rate monitoring and comparison with the standard of care body temperature and heart rate monitoring.

## Methods

### Study design

This observational pilot study was designed to evaluate the accuracy of the wearable in-ear sensor Cosinuss One (Cosinuss Company, Munich). To determine whether this wearable sensor is sufficiently accurate, we performed a comparison with anesthesiologic standard of care monitoring zero-heat flux temperature and urinal bladder monitoring, and heart rate measured by ECG, respectively. Minimalizing the strain on the study participants, especially the continuous body temperature measurement, we chose surgical procedures of non-critical ill patient for this study. Avoiding an influence on bladder temperature measurement by visceral surgery, non-cardiac thoracic procedures were chosen. In order to eliminate potential differences in temperature found between sexes due to female hormone cycle, we enrolled ten male patients in this study. All patients underwent thoracic (non-cardiac) operations including thoracotomy.

Ethical approval for this study was provided by the University of Luebeck Ethics committee (Reg.-Nr.: 16–324). The study was registered in the German Clinical Trials Register (Reg.-No: DRKS00012848). All patients gave their written consent to be included in this observational study. Patients’ demographic and surgical details were recorded.

After introduction of general anesthesia patients received a bladder catheter with temperature monitoring unit (Rüsch Sensor 400, Teleflex Medical Ltd., Ireland). Patients were placed in a lateral position with slight flexion of the torso. All patients received a perioperative warming system under the patient’s body and an additional warming unit on the lower extremities (*MoeckWarmingSystem.* Moeck & Moeck GmbH, Germany). After patient positioning, the Cosinuss One sensor was placed in the external ear canal. The zero-heat flux sensor (*TCore*^*TM*^, Dräger, Germany) was positioned on the skin of the forehead. We confirmed adequate positioning at intercurrent controls throughout the study period. Initial five minutes of measurement were discarded to allow sensors to equilibrate, and measurements were restricted to the operation period.

Vesical bladder temperature, zero-heat flux, and ECG (*Infinity*^*TM*^* Delta Monitor*, Dräger, Germany) were recorded electronically in one-minute intervals by Dräger Infinity monitor. The in-ear temperature and heart rate were recorded electronically in one second intervals wirelessly using the Cosinuss Lab app. Operation theatre temperature was measured at the beginning and ending of each surgical procedure.

After surgery, we removed the zero-heat flux and in-ear sensors and examined the patients’ skin and external ear canal for injuries.

### Wearable Cosinuss One in-ear temperature and heart rate sensor

**Fig. 1:**
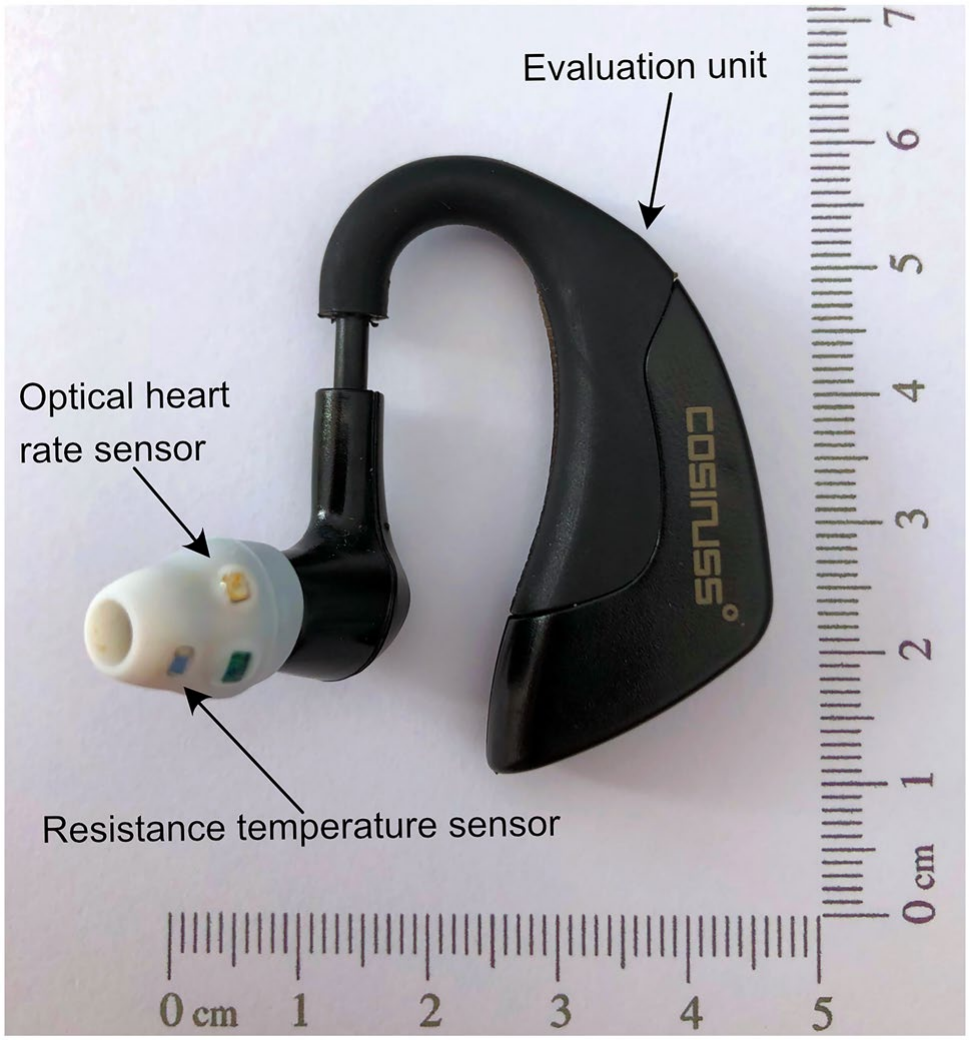
Wearable Cosinuss One in-ear sensor for continuous temperature and heart rate monitoring

The Cosinuss One in-ear sensor is developed and commercially distributed by Cosinuss Company, Munich, Germany. It is composed of a sensor element connected to an evaluation unit behind the ear via a connecting cable that is customized to the anatomy of the external ear (Fig. 1). The size of the sensor is 45 × 38 × 18mm and it weights 6.5 g (Table 1). A resistance thermometer is used for body temperature monitoring with an accuracy of ± 0.2 °C reported by the manufacturer. Using photoplethysmogram (PPG) with green light, the sensor measures the pulse rate in the external auditory canal by means of reflection measurement. Minimizing movements artefacts, the wearable sensor is available in different sizes. The accuracy is ± 1BPM for heart rate monitoring, reported by the manufacturer. The sensor probe is placed in the external auditory canal. The tip of the sensor probe does not touch the tympanic membrane. Recorded data are sent in real-time from the sensor unit to the Cosinuss Lab app wirelessly. After cleaning the Cosinuss One sensor with alcohol-free disinfection wipes, the device can be reused.

**Table 1:** Characteristics of the wearable in-ear sensor Cosinuss One

Overall size	45 × 38 x 18 mm
Overall weight	6.5 g
Operating temperature	-15 to 55 °C
Certification	Bluetooth Low Energy, ANT + , CE
Battery life	approx. 7 h
Resistance temperature monitoring	
Type sensor	Pt1000
Method of measurement	Resistance temperature sensor
Range of measurement	0 to 50 °C
Accuracy	± 0.2 °C
Time resolution	1 Hz
Optical heart rate monitoring	
Method of measurement	Circummission-Method
Range of measurement	35—220 bpm
Accuracy	± 1 bpm
Time resolution	1 Hz

### Data management and statistical analysis

All analyses were performed using the statistical software environment R 3.6.1 [15]. Since the Cosinuss One in-ear sensor measured several times per minute, we averaged the measurements in windows of one minute, centered around the time points of the Dräger^®^ Monitor (zero-heat flux and bladder temperature and ECG heart rate monitoring) measurements. We set a priori acceptable agreement between Cosinuss One and bladder temperature and zero-heat-flux monitoring, respectively, to be 0.5 °C. We chose this limit due to normal human circadian temperature variation and occurrence of clinical complications starting from a temperature difference > 0.5 °C [2, 16, 17]. We set a priori acceptable agreement between Cosinuss One and ECG heart rate monitoring to be 2 BPM.

To compare the paired measurements of the devices, we estimated limits of agreement according to the Bland and Altman analysis method [18]. We used the true value varies extension of the Bland and Altman analysis for repeated measurements [19]. We estimated the bias ($B$) and the standard deviation of the differences of the paired measurements (${\upsigma }_{d}$) to obtain the limits of agreement $\mathrm{LoA}=\mathrm{B}\pm 1.96\cdot {\upsigma }_{\mathrm{d}}$. The confidence interval for $B$ was constructed by assuming a Student’s $t$-distribution with $n-1$ degrees of freedom, where $n$ is the total number of paired measurements. The confidence intervals for the upper and lower $LoA$ were based on a modified mean sum of squares to estimate the between-subject variance proposed by Thomas & Hultquist [20], if the ratio of between-subject variance and total variance is less than 1/3 as suggested by Olofsen et al. [19]. Construction of confidence intervals was performed by the method of variance estimates recovery (MOVER) [21, 22]. Further details on the Bland–Altman analysis can be found in the Supplement.

In addition, we followed an alternative approach based on linear mixed models to estimate limits of agreement, concordance correlation coefficient and the total deviation index while allowing for linear trends of the differences of the paired measurements [23]. Details on this method and the respective results can be found in the Supplement.

## Results

### Patients’ characteristics

In order to eliminate bias of body temperature between sexes, the Cosinuss One was evaluated in ten male patients undergoing non-cardiac thoracic surgical procedures (8 Lobectomy, 1 Pneumonectomy, 1 Pleurectomy + HITHOC). Patients and surgical characteristics are shown in Table 2. Overall surgical time was 190 ± 102 min (range: 102 – 455 min). The wearable sensor was well tolerated in all patients. After surgery and in-ear sensor removal examination of the outer ear canal and auricle showed no tissue damage. None the patients complained any pain of the ear after the surgery.

**Table 2:** Demographic and surgical characteristics

Demographic and surfgical characteristics	
Age (years)	72 ± 9
Weight (kg)	89.1 ± 16.6
Height (cm)	179 ± 9,5
Body mass index (kg/m^2^)	27.7 ± 4.3
Surgical Times	190 ± 102
Type Surgery	
*Lobectomy*	8
*Pneunonectomy*	1
*Other*	1

### Temperature monitoring

**Fig. 2:**
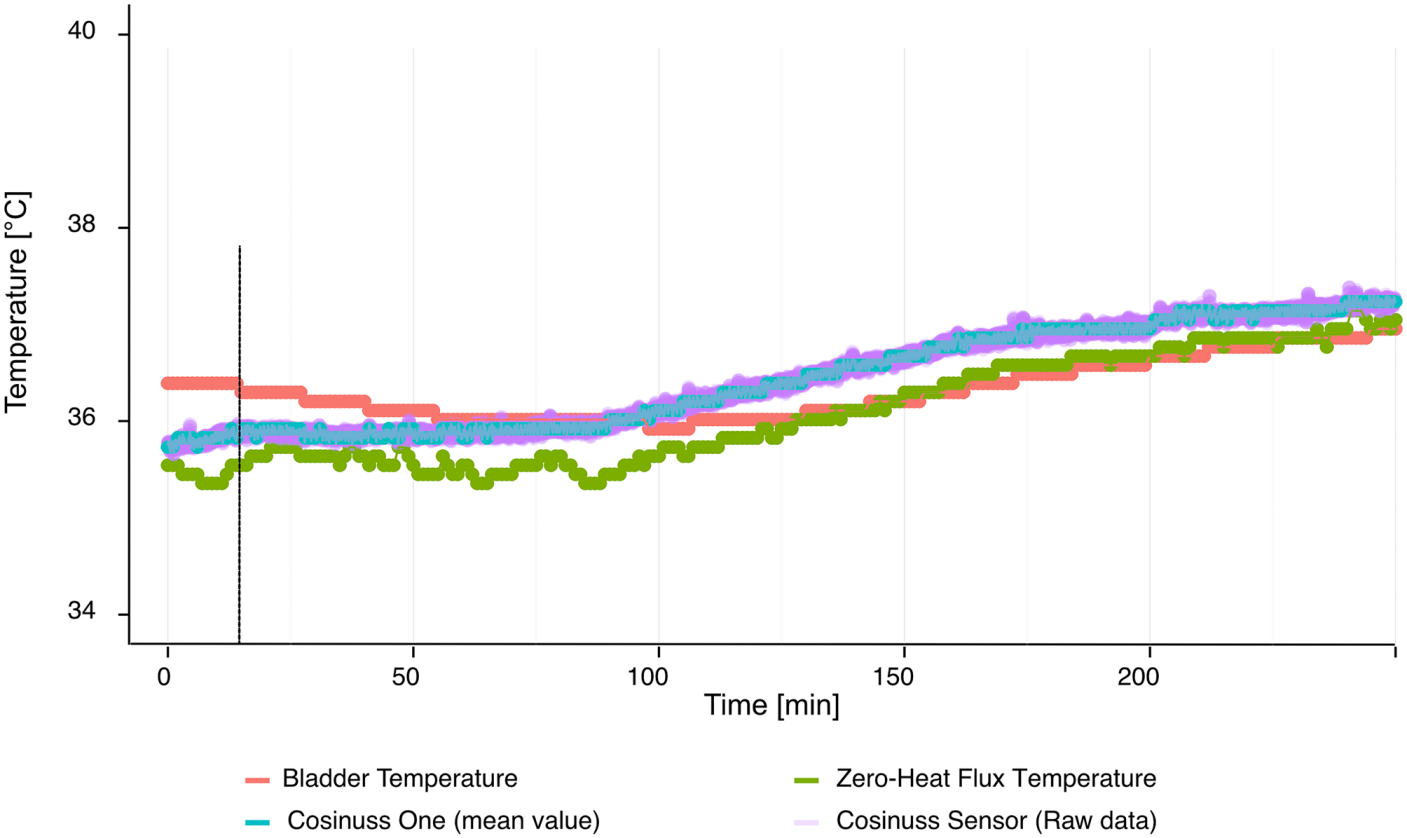
Exemplary presentation of temperatures curves. The Cosinuss One sensor (mean value) is depicted as the cyan line, the Cosinuss One raw data are depicted as the pink line, the bladder temperature monitoring is depicted as the red line and the zero-heat flux temperature monitoring is depicted as the green line. Cosinuss One and zero-heat flux monitoring needed a short equilibration period (vertical dotted black line). Bladder temperature monitoring showed always higher temperatures at the beginning of surgery

Temperature data were obtained from all patients. In one case zero-heat-flux temperature monitoring was lost due to storage failure of the Dräger monitor. Zero-heat-flux (T_B_) and Cosinuss One (T_C_) temperature sensor needed a short time of equilibration. Bladder temperature monitoring (T_U_) showed a period of decrease of temperature during the first minutes of surgery and more slowly temperature increase than zero-heat-flux and Cosinuss One monitoring (Fig. 2). A total of 1423 pairs of T_C_ and T_U,_ and 1288 pairs of T_C_ and T_B_ were collected, ranging from 34.8 °C to 38.2 °C.

### Cosinuss One (TC) vs. bladder temperature (TU) monitoring

The Bland–Altman analysis (Table 3) estimated an average bias of -0.15 °C (values of T_U_ are lower on average than T_C_ temperature readings, 95% confidence interval [-0.3, -0.002]) between T_U_ and T_C_, suggesting a significant difference on average. 95% of the readings are in the range of [-0.79, 0.49] (LoA) with 95% confidence intervals [-1.03, -0.65] (lower LoA) and [0.35, 0.73] (upper LoA). The range between the lower confidence bound of the lower LoA and the upper confidence bound of the upper LoA is 1.76 °C, greater than the pre-defined acceptable limit of agreement of ± 0.5 °C (Fig. 4A and Table 3). 89% of measurement pairs have an absolute difference ≤ 0.5 °C. The within-subject variance is estimated as 0.08 ± 0.003, higher than 0.03 ± 0.01, the between-subject variance. These results were supported by the alternative analysis (results shown in Supplement).

**Table 3:** Bland–Altman analysis results

	***Cosinuss Sensor (T***_***C***_***) vs. bladder temperature (T***_***U***_***) monitoring***	***Cosinuss Sensor (T***_***C***_***) vs. brain temperature (T***_***B***_***) monitoring***	***Cosinuss Sensor (H***_***C***_***) vs. ECG heart rate monitoring (H***_***D***_***)***
**Bias ± S.E**	-0.1517 ± 0.0648	-0.2172 ± 0.0774	-0.2720 ± 0.1056
**95% Confidence Interval of bias**	[-0.3012; -0.0022]	[-0.3957; -0.0388]	[-0.5109; -0.0332]
**SD of differences ± S.E**	0.3258 ± 0.0229	0.2865 ± 0.0471	2.3153 ± 0.0407
**Limits of Agreement (LoA)**	[-0.7903; 0.4869]	[-0.7788; 0.3444]	[-4.8101; 4.2660]
**95% Confidence Interval of lower LoA**	[-1.0303; -0.6527]	[-1.1789; -0.5906]	[-5.0856; -4.5555]
**95% Confidence Interval of upper LoA**	[0.3493; 0.7270]	[0.1561; 0.7444]	[4.0115; 4.5416]
**Within-subject variance (WSV) ± S.E**	0.0773 ± 0.0028	0.0285 ± 0.0010	5.2980 ± 0.1840
**Between-subject variance (BSV) ± S.E**	0.0288 ± 0.0147	0.0537 ± 0.0269	0.0628 ± 0.0450
**Ratio of BSV and total variance (**${\varvec{\uptau}}$**) ± S.E**	0.2717 ± 0.1008	0.6535 ± 0.1140	0.0117 ± 0.0083
**Modified analysis method**^**a**^	No	Yes	No

^a^The modified analysis method by Thomas and Hultquist [14] is used if the ratio between between-subject variance and total variance is greater than 1/3

The diagnostic plots (Fig. 5 in Supplement) show no major deviations from the underlying assumptions (normality of differences, constant variability). However, the higher absolute differences for higher temperatures indicate a linear trend of the bias. Thus, the LoA based on the Bland–Altman analysis may not be adequate.

### Cosinuss One (TC) vs. Dräger^®^ TCore (TB) temperature monitoring

The Bland–Altman (Table 3) analysis estimates an average bias of -0.22 °C (values of *T*_*B*_ are lower on average than *T*_*C*_temperature readings, 95% confidence interval [-0.40, -0.04]) between *T*_*B*_ and *T*_*C*_, suggesting a significant difference on average. 95% of the readings are in the range of [-0.78, 0.34] (LoA) with 95% confidence intervals [-1.18, -0.59] (lower LoA) and [0.16, 0.74] (upper LoA). The range between the lower confidence bound of the lower LoA and the upper confidence bound of the upper LoA is 1.92, greater than the pre-defined acceptable limit of agreement of ± 0.5 °C (see Fig. 4B and Table 3). 79% of measurement pairs have an absolute difference ≤ 0.5 °C. The within-subject variance is estimated as 0.03 ± 0.001, lower than 0.05 ± 0.03, the between-subject variance. These results were supported by the alternative analysis (results shown in Supplement).

Again, the diagnostic plots (Fig. 6 in Supplement) show no major deviations from the underlying assumptions (normality of differences, constant variability). However, the higher absolute differences for higher temperatures indicate a linear trend of the bias. Thus, the LoA based on the Bland–Altman analysis may not be adequate.

### Heart rate

#### Cosinuss One (HC) vs. ECG (HD) heart rate monitoring

**Fig. 3:**
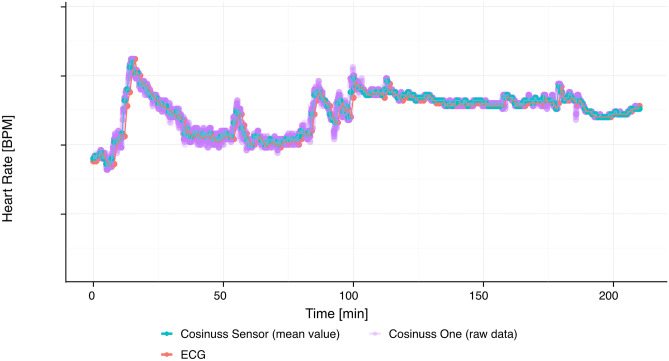
Exemplary presentation of heart rate curves. The Cosinuss One sensor mean value are depicted as the cyan line, the Cosinuss One raw data are depicted as the pink line and the ECG heart rate monitoring is depicted as the red line

**Fig. 4:**
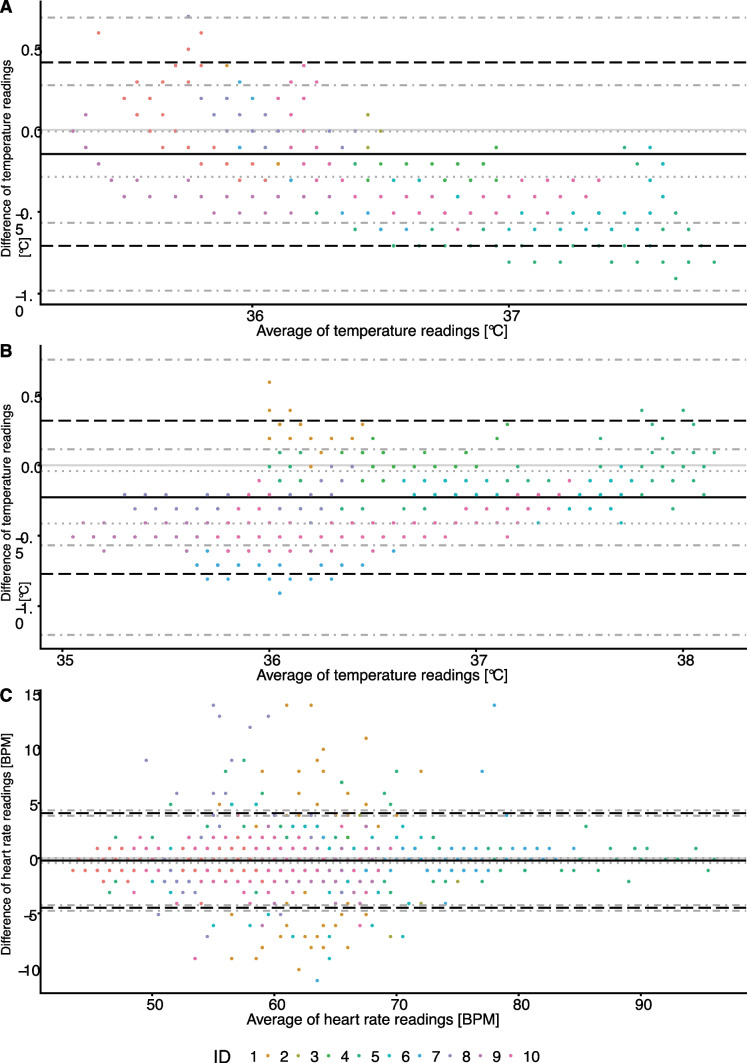
Bland–Altman plots of temperature measurements of **A** bladder (T_U_) and in-ear sensor (T_C_), **B** zero heat flux (T_B_) and in-ear sensor (T_C_), and **C** heart rate measurements. Each point depicts one paired measurement of the respective methods. Repeated pairs of measurements of one patient are shown in the same color. The solid line highlights the bias with 95% confidence interval shown as dotted lines. Limits of agreement are shown as dashed lines with respective 95% confidence intervals shown as dash-dotted lines

There were between 48 and 456 paired measurements over time by the Cosinuss One (H_C_) and the ECG heart rate monitoring (H_D_) on each sample (Fig. 3), ranging from 43 to 96 BPM. The Bland–Altman analysis (Table 3) estimates an average bias of -0.27 BPM (values of Dräger Monitor are lower on average than Cosinuss Sensor readings, 95% confidence interval [-0.51, -0.03]) between Dräger Monitor and Cosinuss Sensor, suggesting a significant difference on average. 95% of the readings are in the range of [-4.81, 4.27] (LoA) with 95% confidence intervals [-5.09, -4.56] (lower LoA) and [4.01, 4.54] (upper LoA). The range between the lower confidence bound of the lower LoA and the upper confidence bound of the upper LoA is 9.63, greater than the pre-defined acceptable limit of agreement of 2 BPM (Fig. 4C and Table 3). 84% of measurement pairs have an absolute difference ≤ 2 BPM. The within-subject variance is estimated as 5.30 ± 0.18, higher than 0.06 ± 0.05, the between-subject variance. These results were supported by the alternative analysis (results shown in Supplement).

The diagnostic plots (Fig. 7 in Supplement) show some deviation from the underlying assumption of normality of differences. Thus, the LoA based on the Bland–Altman analysis may not be adequate.

## Discussion

In this study, we were able to show that the wearable in-ear sensor offers reliable continuous body temperature and heart rate monitoring. Focusing on comparable monitoring sites and reducing patients’ stress, we will discuss the study results in the context of intraoperative body temperature and heart rate monitoring as well.

Observing body temperature by bladder, zero-heat flux and Cosinuss One sensor monitoring, three aspects were noticeable. First, the Cosinuss One enabled a reliable continuous measurement of body temperature. We could not detect outliers of temperature monitoring. Second, we found an inter-subject variation of temperature curves measured by all three devices. This inter-subject variation can be assumed by circadian rhythm, individual thermoregulation, and impairment of thermoregulatory control due to anesthetic drugs [24, 25]. Sessler reviewed that in the first period after induction of general anesthesia body temperature decreased significantly due to anesthetic-induced vasodilation [24]. Accordingly, in our observation, bladder temperature monitoring revealed a decrease at the beginning of surgery. We assume that all patients had a decrease of body temperature after induction of general anesthesia which stopped after warming patient’s body. However, bladder temperature showed a lag of responsiveness. A review about bladder temperature monitoring by Fallis supports our assumption [26]. However, this site of temperature monitoring is an useful indicator of total body rewarming [27]. Warming all patients during surgery, temperature monitoring of all sensors showed an increase of body temperature. Last, due to the kind of temperature measurement, zero-heat flux and in-ear temperature monitoring needed an equilibration.

The limits of agreement of Cosinuss One vs. bladder temperature monitoring and Cosinuss One vs. zero-heat flux temperature monitoring, respectively, were greater than the a priori acceptable limit of 0.5 °C. A closer look reveals that T_B_ and T_U_ also vary by more than 0.5 °C (see supplement). Comparing zero-heat flux temperature monitoring to urinary or pulmonary artery catheter temperature monitoring site, several studies also reported a temperature difference > 0.5 °C. In these studies, the authors argued that a high proportion of differences supported a sufficient agreement of the sensors [28–30]. In this respect, it must be considered that different temperature measuring methods and different measurement sites are also included in the comparison. As a result, the temperature site remains often part of the discussion in the literature. In order to establish a classification of the non-invasive sensors, we have used two non-invasive or minimally invasive measuring methods and locations.

Selecting bladder and zero-heat flux thermometry for reference temperature monitoring sites, we focused on the avoidance of patients’ stress and comparable monitoring sites. Zero-heat flux temperature monitoring was well tolerated by the study participants [29]. Every patient per protocol got a bladder catheter with temperature monitoring undergoing extended thorax surgery (e.g., lobectomy). Thus, there was no additional stress for the patients. Several studies comparing zero-heat flux and bladder temperature monitoring to invasive methods (e.g., pulmonary artery catheter, esophageal probe, arterial catheter) showed comparable results of body temperature [2, 26, 29, 30]. The advantage of bladder temperature monitoring during thoracic surgery is an intact abdomen. There is no impairment of measurement. In contrast, esophageal body temperature monitoring might be impaired by open chest and lavage during surgery [31].

In the light of the above, the inaccuracy of the sensors has to be considered in addition to the a priori limit. Additionally, in 89% of measurements, in-ear temperature monitoring was within 0.5 °C of bladder temperature monitoring. 78.87% of measurements were within the priori limit of 0.5 °C of zero-heat flux monitoring. Considering that bladder temperature monitoring site is more valid than zero-heat flux as body core temperature monitoring site, our results appear to reasonably estimate body temperature.

Our results show a reliable agreement of in-ear heart rate monitoring and ECG. ECG is the gold standard of heart rate monitoring during anesthesia, surgery, and intensive care. Several studies showed a good agreement between PPG in-ear heart rate monitoring and ECG [32, 33]. Using green spectrum of light, the Cosinuss One sensor receives a stronger PGG signal. Passler et al. showed good levels of agreement between Cosinuss One and ECG heart rate monitoring [10]. Additionally, several studies showed that wearable technologies enabled detection of cardiac events [34, 35]. Breteler et al. concluded in their study that wearable continuous monitoring may have the potential to contribute to early recognition of physiological decline in high-risk patients [36].

Our study has a few limitations. First, we enrolled a small number of patients. The University of Luebeck Ethics committee only allowed to evaluate ten male patients. A strength, on the other hand, is that each patient was analyzed in a standardized set-up due to the surgical and anesthesia standards for minimum 45 min, yielding up to 1423 pairs of body temperature and 456 pairs of heart rate monitoring, respectively. Second, we did not evaluate the wearable sensor during rapid body temperature changes. Several studies showed that there is a greater bias of zero-heat flux and bladder temperature monitoring during rapid body temperatures changes (e.g., hypothermia during cardiopulmonary bypass, HIPEC) [37, 38]. Both reference temperature monitoring sites are not designed for rapid body temperature changes monitoring. Second, the in-ear sensor measured body temperature and heart rate once a second. Bladder and zero-heat flux temperature monitoring were recorded only once a minute due to manufacture determined monitoring setting. There might be a greater bias between the in-ear sensor and bladder and zero-heat flux temperature monitoring, respectively, because we estimated the minute means of body temperature and heart rate recorded by the in-ear sensor. Finally, we compared the in-ear sensor with the ECG. But each ECG signal has not be converted into a pulse wave during an extra-systole.

The advantage of this wearable sensor is the continuous body temperature and heart rate monitoring. It is well tolerated by patients and offers a non-invasive monitoring, especially for outpatient monitoring. The advantage of wearable sensors is monitoring clinical parameters without interfering with the current activity. Body temperature and heart rate monitoring are two cornerstones of detection of critical illness in COVID-19 patients. Oxygen saturation is the third cornerstone completing the wearable non-invasive monitoring. All three parameters are important to detect the silent hypoxia in Covid-19 pneumonia and the shift from non-critical illness to critical illness. Due to the COVID-19 pandemic several studies evaluate the application of wearable non-invasive devices [1, 39]. The next generation of the Cosinuss sensors will allow for monitor oxygen saturation (Cosinuss Two sensor). A clinical study initiated by Schmidt et al. at the Klinikum rechts der Isar, Munich, investigates the Cosinuss Two sensor monitoring in COVID-19 outpatients (Tele-COVID study – Remote Patient Monitoring for Covid-19) [40].

In conclusion, the Cosinuss One sensor enables a good validity of body temperature and heart rate monitoring used in the ear. Body temperature and heart rate were reliably measured by the wearable in-ear sensor compared to clinical standard of care monitoring devices. In this context, this observational study is one of the first with respect to measurement accuracy and precision of wearable sensors compared to standard and certificated clinical monitoring devices. Wearable sensors are promising technique that enables out-patient monitoring. The COVID-19 pandemic demonstrated the need of such monitoring devices for monitoring non-critical ill patients and detecting patients with threatening respiratory deterioration. In this regard, we think that guidelines and general standards for wearable sensors toned to be introduced.

## Supplementary Information

Below is the link to the electronic supplementary material.

Supplementary file1 (DOCX 1316 KB)

## Abbreviations

BPM: Beats per minute
HITHOC: Hyperthermic Intrathoracic Chemotherapy
H_c_: Heart rate in-ear sensor
H_D_: Heart rate Dräger monitor (ECG)
LoA: Limits of Agreement
PPG: Photoplethysmogram
T_U_: Urinary bladder temperature
T_B_: Zero-heat flux brain temperature
T_C_: In-ear sensor temperature
