# Supplementary material for: Evaluation of a Wearable in-Ear Sensor for Temperature and Heart Rate Monitoring: A Pilot Study
Source: J Med Syst. 2022 Nov 4;46(12):91. doi: 10.1007/s10916-022-01872-6 (PMC9633487; doi:10.1007/s10916-022-01872-6)
Supplement: Supplementary file 1 — Supplementary file1 (DOCX 1316 KB) [file 10916_2022_1872_MOESM1_ESM.docx]

Validation Study of non-invasive ear-sensor for Temperature and Heart rate monitoring - Supplement

# Statistical Analyses

## Notation

Let measurements from a device $A$ be $Y^{A}$. These are obtained from $i=1,\ldots,n$ subjects denoted by $Y_{i}^{A}$. Multiple measurements $m_{i}$from subject $i$ are indicated by a second subscript $j=1,\ldots,m_{i}$. Thus, a total of $N=\sum_{i=1}^{n} m_{i}$ is available. A model for a single measurement is $Y_{\mathrm{ij}}^{A}=T_{\mathrm{ij}}^{A}+\tilde{E}_{\mathrm{ij}}$, where $T_{\mathrm{ij}}^{A}$ is the true value for subject $i$ at time point $j$ measured by device $A$, and $\tilde{E}_{\mathrm{ij}}^{A}$ is the measurement error. This error can be decomposed into a constant systematic bias $B^{A}$ of device $A$, a random individual error $I_{i}^{A}$, and a random error $E_{\mathrm{ij}}^{A}$. Here, $I^{A}$ and $E^{A}$ are statistically independent random variables with means of zero and variances $\sigma_{A, I}^{2}$ (between-subjects) and $\sigma_{A, w}^{2}$ (within-subject), respectively.

In our setting, $T_{\mathrm{ij}}^{A}$ will vary over the measurement time points $j$.

## Bland-Altman Analysis

Bland and Altman (2007) proposed to evaluate the agreement between two devices $A$ and $B$ by the differences $D_{\mathrm{ij}}=Y_{\mathrm{ij}}^{A}-Y_{\mathrm{ij}}^{B}=T_{\mathrm{ij}}+B+I_{i}+E_{\mathrm{ij}}$ of two sets of measurements $Y^{A}$ and $Y^{B}$, with $T_{\mathrm{ij}}=T_{\mathrm{ij}}^{A}-T_{\mathrm{ij}}^{B}$, $B=B^{A}-B^{B}$, $I_{\mathrm{ij}}=I_{i}^{A}-I_{i}^{B}$, and $E_{\mathrm{ij}}=E_{\mathrm{ij}}^{A}-E_{\mathrm{ij}}^{B}$. The between-subject and within-subject variances of $D$ are given by $\sigma_{D,I}^{2}=\sigma_{A,I}^{2}+\sigma_{B,I}^{2}$ and $\sigma_{D,w}^{2}=\sigma_{A,w}^{2}+\sigma_{B,w}^{2}$. Thus, the total variance $\sigma_{D}^{2}$ of $D$ is given by $\sigma_{D,I}^{2}+ \sigma_{D,w}^{2}$. It is assumed that $T_{\mathrm{ij}}^{A}$ and $T_{\mathrm{ij}}^{B}$ are paired, i.e. $T_{\mathrm{ij}}^{A}-T_{\mathrm{ij}}^{B}=0$.

A Bland-Altman plot shows the differences $D_{\mathrm{ij}}$ versus the means $M_{\mathrm{ij}}=\left( Y_{\mathrm{ij}}^{A}+Y_{\mathrm{ij}}^{B} \right)/2$. The $100\left( 1-\alpha\right)\%$ limits of agreement (LoA) between the two devices are added to the Bland-Altman plot and are defined as $LoA=B\pm z_{\alpha/2}\sigma_{D}$, where $z_{\alpha}$ is the $100\alpha\%$ quantile of a standard normal distribution, assuming that the differences are normally distributed and $B$ and $\sigma_{D}$ are known. In application these have to be estimated.

### Point estimates

As Olofsen et al. (2015) note, the estimation of bias and variances is based on the ANOVA-like mean sums of squares

$$\begin{matrix} MSSR & =\frac{1}{N-n}\sum_{i=1}^{n} \sum_{j=1}^{m_{i}} \left( D_{ij}-\hat{B}_{i} \right)^{2}, \\ MSSI & =\frac{1}{n-1}\sum_{i=1}^{n} m_{i}\left( \hat{B}_{i}-\hat{B} \right)^{2}, \end{matrix}$$

where $\hat{B}=\frac{1}{N}\sum_{i=1}^{n} \sum_{j=1}^{m_{i}} D_{ij}$, and $\hat{B}_{i}=\frac{1}{m_{i}}\sum_{k=1}^{m_{i}} D_{ik}$. If the intraclass correlation $\tau=\frac{MSSI-MSSR}{MSSI+\lambda_{1} MSSR}$, $\lambda_{1}=\left( 1-\frac{N^{2}-\sum_{i=1}^{n} m_{i}^{2}}{N\left( n-1 \right)} \right)$ is less than $1/3$, the variance of the differences is estimated in a standard way. Otherwise, the modified way of Thomas and Hultquist (1978) is used.

#### Standard method

The bias $B$ is estimated by $\hat{B}=\frac{1}{N}\sum_{i=1}^{n} \sum_{j=1}^{m_{i}} D_{\mathrm{ij}}$.

Following the usual ANOVA approach, estimates for $\sigma_{D,w}^{2}$ and $\sigma_{D,I}^{2}$

$$\begin{matrix} \hat{\sigma}_{D,w}^{2} & =MSSR, \\ \hat{\sigma}_{D,I}^{2} & =\frac{MSSI-MSSR}{\lambda_{1}}. \end{matrix}$$

Thus, $\hat{\sigma}_{D}^{2}=\hat{\sigma}_{D,w}^{2}+\hat{\sigma}_{D,I}^{2}=\frac{1}{\lambda_{1}}MSSI+\left( 1-\frac{1}{\lambda_{1}} \right)MSSR$.

#### Modified method

The bias $B$ is estimated by $\hat{B}_{a}=\frac{1}{n}\sum_{i=1}^{n} \frac{1}{m_{i}}\sum_{j=1}^{m_{i}} D_{\mathrm{ij}}$.

Thomas and Hultquist (1978) modified the calculation of the $MSSI$ by not using the grand mean but the mean of the individual means, i.e. the bias estimate $\hat{B}_{a}$:

$$MSSI_{a}=\frac{1}{n-1}\sum_{i=1}^{n} \left( \hat{B}_{i}-\hat{B}_{a} \right)^{2}.$$

This results in estimates for $\sigma_{D,w}^{2}$ and $\sigma_{D,I}^{2}$ given by

$$\begin{matrix} \hat{\sigma}_{D,w}^{2} & =MSSR, \\ \hat{\sigma}_{D,I_{a}}^{2} & =MSSI_{a}-\frac{1}{n}\sum_{i=1}^{n} \frac{1}{m_{i}}MSSR. \end{matrix}$$

Thus, $\hat{\sigma}_{D_{a}}^{2}=\hat{\sigma}_{D,w}^{2}+\hat{\sigma}_{D,I_{a}}^{2}=MSSI+\left( 1-\frac{1}{n}\sum_{i=1}^{n} \frac{1}{m_{i}} \right)MSSR=MSSI+\lambda_{2}MSSR$.

### Confidence intervals

#### Confidence interval for the bias

##### Standard method

The $100\left( 1-\alpha\right)\%$ confidence interal for $\hat{B}$ can be constructed under the assumption of normallity of $D$ by

$$\hat{B}\pm t_{1-\alpha/2,n-1}\hat{\sigma}_{\hat{B}}^{2}.$$

Here, $\hat{\sigma}_{\hat{B}}^{2}=\frac{\hat{\sigma}_{D,w}^{2}}{N}+\frac{\sum_{i=1}^{n} m_{i}^{2}}{N^{2}}\hat{\sigma}_{D,I}^{2}$, and $t_{\alpha,f}$ is the $100\alpha\%$ quantile of a Student’s $t$ distribution with degrees of freedom $f$.

##### Modified method

The $100\left( 1-\alpha\right)\%$ confidence interal for $\hat{B}$ can be constructed under the assumption of normallity of $D$ by

$$\hat{B}_{a}\pm t_{1-\alpha/2,n-1}\hat{\sigma}_{\hat{B}_{a}}^{2}.$$

Here, $\hat{\sigma}_{\hat{B}_{a}}^{2}=\frac{1}{n^{2}}\sum_{i=1}^{n} \frac{1}{m_{i}}\hat{\sigma}_{D,w}^{2}+\frac{1}{n}\hat{\sigma}_{D,I}^{2}$, and $t_{\alpha,f}$ is the $100\alpha\%$ quantile of a Student’s $t$ distribution with degrees of freedom $f$.

#### Confidence intervals for the limits of agreement

Special attention has to be payed to confidence intervals for the limits of agreement. These are constructed by the Methods of Variance Estimates Recovery (MOVER). According to Zou (2013), the $100\left( 1-\beta\right)\%$ confidence intervals for the lower ($l$) and upper ($u$) LoA are given by $\mathrm{Lo}A_{l}-LME$, $\mathrm{Lo}A_{l}+RME$, $\mathrm{Lo}A_{u}-LME$, and $\mathrm{Lo}A_{u}+RME$

##### Standard method

For the standard method, $LME$ and $RME$ are given by

$$\begin{matrix} LME & =\sqrt{z_{\beta/2}^{2}\hat{\sigma}_{\hat{B}}^{2}+z_{\alpha/2}^{2}\left( \sqrt{u}-\sqrt{\hat{\sigma}_{D}^{2}} \right)^{2},} \\ RME & =\sqrt{z_{\beta/2}^{2}\hat{\sigma}_{\hat{B}}^{2}+z_{\alpha/2}^{2}\left( \sqrt{\hat{\sigma}_{D}^{2}}-\sqrt{l} \right)^{2}}, \end{matrix}$$

with

$$\begin{matrix} u & =\hat{\sigma}_{D}^{2}+\sqrt{\left( \frac{MSSI-u_{1}}{\lambda_{1}} \right)^{2}+\left( \left( 1-\frac{1}{\lambda_{1}} \right)\left( MSSR-u_{2} \right) \right)^{2},} \\ l & =\hat{\sigma}_{D}^{2}-\sqrt{\left( \frac{MSSI-l_{1}}{\lambda_{1}} \right)^{2}+\left( \left( 1-\frac{1}{\lambda_{1}} \right)\left( MSSR-l_{2} \right) \right)^{2}}, \end{matrix}$$

where $l_{1}=\frac{MSSI\left( n-1 \right)}{\chi_{1-\beta/2,n-1}^{2}}$, $u_{1}=\frac{MSSI\left( n-1 \right)}{\chi_{\beta/2,n-1}^{2}}$, $l_{2}=\frac{\left( N-n \right)MSSR}{\chi_{1-\beta/2,N-n}^{2}}$, $u_{2}=\frac{\left( N-n \right)MSSR}{\chi_{\beta/2,N-n}^{2}}$, and $\chi_{\beta,f}^{2}$ is the $100\beta\%$ quantile of the chi-square distribution with degrees of freedom $f$.

##### Modified method

For the modified method, $LME$ and $RME$ are given by

$$\begin{matrix} LME & =\sqrt{z_{\beta/2}^{2}\hat{\sigma}_{\hat{B}_{a}}^{2}+z_{\alpha/2}^{2}\left( \sqrt{u}-\sqrt{\hat{\sigma}_{D}^{2}} \right)^{2}}, \\ RME & =\sqrt{z_{\beta/2}^{2}\hat{\sigma}_{\hat{B}_{a}}^{2}+z_{\alpha/2}^{2}\left( \sqrt{\hat{\sigma}_{D}^{2}}-\sqrt{l} \right)^{2}}, \end{matrix}$$

with

$$\begin{matrix} u & =\hat{\sigma}_{D}^{2}+\sqrt{\left( MSSI_{a}-u_{1} \right)^{2}+\left( \left( 1-\lambda_{2} \right)\left( MSSR-u_{2} \right) \right)^{2}}, \\ l & =\hat{\sigma}_{D}^{2}+\sqrt{\left( MSSI_{a}-l_{1} \right)^{2}+\left( \left( 1-\lambda_{2} \right)\left( MSSR-l_{2} \right) \right)^{2}}, \end{matrix}$$

where $l_{1}=\frac{MSSI\left( n-1 \right)}{\chi_{1-\beta/2,n-1}^{2}}$, $u_{1}=\frac{MSSI\left( n-1 \right)}{\chi_{\beta/2,n-1}^{2}}$, $l_{2}=\frac{\left( N-n \right)MSSR}{\chi_{1-\beta/2,N-n}^{2}}$, $u_{2}=\frac{\left( N-n \right)MSSR}{\chi_{\beta/2,N-n}^{2}}$, and $\chi_{\beta,f}^{2}$ is the $100\beta\%$ quantile of the chi-square distribution with degrees of freedom $f$.

## Linear Mixed Model Analysis

If the differences of measurements follow a linear trend, standard Bland-Altman analysis may not be appropriate Carstensen (2010). An alternative approach which can handle these trends is proposed by Choudhary and Nagaraja (2017) and based on a linear mixed model.

As Choudhary and Nagaraja (2017) write, our data are linked repeated measurements. In this case, the measurements by the two devices to be compared are modelled by mixed-effects models

$$Y_{ij}^{A}=T_{i}+I_{i}^{A}+\tilde{T}_{ij}+E_{ij}^{A},$$

and

$$Y_{ij}^{B}=\beta_{0}+T_{i}+I_{i}^{B}+\tilde{T}_{ij}+E_{ij}^{B}.$$

Here, opposed to the model from the definition above, is assumed, that each individual $i$ has a true mean unabservable measurement $T_{i}$ and there is a subject$\times$time interaction $\tilde{T}_{ij}$ which follows an independent $\mathcal{N}\left( 0,\sigma_{\tilde{T}}^{2} \right)$. In contrast to $I_{i}^{k}$, which models the effect of device $k$ on measurements for subject $i$, $\tilde{T}_{ij}$ does not depend on the method and cpatures the change in the true value of the $i$th subject over time. However, both effects depend on the subject. The other random variables are assumed to be distributed as follows and mutually independent:

- $T_{i}\sim\mathcal{N}\left( \mu_{T},\sigma_{T}^{2} \right)$
- $I_{i}^{k}\sim\mathcal{N}\left( 0,\psi^{2} \right)$, $k\in\left\{ A,B \right\}$
- $E_{ij}^{k}\sim\mathcal{N}\left( 0,\sigma_{E_{j}^{k}}^{2} \right)$, $k\in\left\{ A,B \right\}$

The term $\beta_{0}$ is the difference in the fixed biases of the two devices.

Taken together, the $m_{i}$ measurements on subject $i=1,\ldots,n$ are independent $m_{i}$-variate normals with means $\mathbb{E}\left( Y_{ij}^{A} \right)=\mu_{T}$ and $\mathbb{E}\left( Y_{ij}^{B} \right)=\beta_{0}+\mu_{T}$, variances $\mathbb{V}ar\left( Y_{ij}^{k} \right)=\sigma_{T}^{2}+\psi^{2}+\sigma_{\tilde{T}}^{2}+\sigma_{E_{j}^{k}}^{2}$ and covariances $\mathbb{C}ov\left( Y_{ij_{1}}^{k},Y_{ij_{2}}^{k} \right)=\sigma_{T}^{2}+\psi^{2}$, $j_{1}\neq j_{2}$ (covariance between two measurements from the same device), $\mathbb{C}ov\left( Y_{ij}^{A},Y_{ij}^{B} \right)=\sigma_{T}^{2}+\sigma_{\tilde{T}}^{2}$ (covariance between two devices at the same time), and $\mathbb{C}ov\left( Y_{ij_{1}}^{A},Y_{ij_{2}}^{B} \right)=\sigma_{T}^{2}$, $j_{1}\neq j_{2}$ (covariance between two measurements from two devices).

Now, the common distribution of a single measurement pair $\left( Y^{A},Y^{B} \right)$ is given by

$$\left( \begin{matrix} Y^{A} \\ Y^{B} \end{matrix} \right)\sim\mathcal{N}_{2}\left( \left( \begin{matrix} \mu_{T} \\ \beta_{0}+\mu_{T} \end{matrix} \right),\left( \begin{matrix} \sigma_{T}^{2}+\psi^{2}+\sigma_{\tilde{T}}^{2}+\sigma_{E^{A}}^{2} & \sigma_{T}^{2}+\sigma_{\tilde{T}}^{2} \\ \sigma_{T}^{2}+\sigma_{\tilde{T}}^{2} & \sigma_{T}^{2}+\psi^{2}+\sigma_{\tilde{T}}^{2}+\sigma_{E^{B}}^{2} \end{matrix} \right) \right).$$

Thus, the differences of two measurements are distributed as

$$D\sim\mathcal{N}\left( \beta_{0},2\psi^{2}+\sigma_{E^{A}}^{2}+\sigma_{E^{B}}^{2} \right)$$

This model can be fit with a fixed effect for the device, and random intercept and random effects for method and measurement time point per subject. From this we get maximum likelihood estimates $\left( \hat{\beta}_{0},\hat{\mu}_{T},\hat{\sigma}_{T}^{2},\hat{\psi}^{2},\hat{\sigma}_{E^{A}}^{2},\hat{\sigma}_{E^{B}}^{2} \right)$. Their standard errors are calculated by inverting the respective Hessian and large-sample theory is used to compute Wald type confidence bounds and intervals.

The mean measurement values are given by $\hat{\mu}_{T}$ and $\hat{\mu}_{T}+\hat{\beta}_{0}$ for devices $A$ and $B$, respectively. The estimated distribution of $D$ is used to compute the limits of agreement as $LoA=\hat{\beta}_{0}\pm z_{1-\alpha/2}\sqrt{2\hat{\psi}^{2}+\hat{\sigma}_{E^{A}}^{2}+\hat{\sigma}_{E^{B}}^{2}}$.

The concordance correlation coefficient ($CCC$) and the total deviation index ($TDI$) are calculated by

$$\begin{matrix} CCC & =\frac{2\left( \hat{\sigma}_{T}^{2}+\hat{\sigma}_{\tilde{T}}^{2} \right)}{\hat{\beta}_{0}^{2}+2\left( \hat{\sigma}_{T}^{2}+\hat{\psi}^{2}+\hat{\sigma}_{\tilde{T}}^{2} \right)+\hat{\sigma}_{E^{A}}^{2}+\hat{\sigma}_{E^{B}}^{2}}, \\ TDI_{p} & =\sqrt{\left( 2\hat{\psi}^{2}+\hat{\sigma}_{E^{A}}^{2}+\hat{\sigma}_{E^{B}}^{2} \right)\chi_{p,1}^{2}\left( \frac{\hat{\beta}_{0}^{2}}{2\hat{\psi}^{2}+\hat{\sigma}_{E^{A}+\hat{\sigma}_{E^{B}}}^{2}} \right)}. \end{matrix}$$

For details on estimation and construction of confidence bands and intervals we refer to Choudhary and Nagaraja (2017).

The $CCC$ can be interpreted as the extent of agreement in excess of what is expected by chance alone and is bounded between -1 and 1. Large positive values of CCC indicated good agreement between the two measurements, with $CCC$=1 implying perfect agreement ($\mu_{1}=\mu_{2},\sigma_{1}=\sigma_{2},\rho=1)$. The $TDI$ is based on the statistical properties of the difference $D$ between two measurements. However, it does not require the normality assumption for $D$ as the Limits of Agreement (LoA) in Bland-Altman analyses do. The $TDI$ has to be calculated for a parameter $p$ and reflects the 100$p$th percentile of $\left| D \right|$. Thus, if $TDI_{p}=0$ for all $p$, the methods have perfect agreement. In this study, we used ${TDI}_{0.9}$, i.e. the difference that 90% of the measurements do not exceed.

### Results

#### Cosinuss One (T_C_) vs. bladder temperature (T_U_) monitoring

The estimated means were 36.25 °C (95% confidence interval [35.96, 36.54]) and 36.31 °C (95% confidence interval [36.17, 36.44]) for T_U_ and T_C_, respectively. The variance of T_U_ was 0.2898 and the variance of T_C_ was 0.3599. Their differences had mean 0.06 °C (values of T_U_ are lower on average than T_C_ temperature readings) and standard deviation 0.3492. Thus 95% LoA were [-0.63, 0.74]. For evaluation of agreement, the estimates and 95% confidence bounds were, respectively, 0.8052 and 0.6758 for $CCC$, and 0.5821 and 0.6711 for ${TDI}_{0.9}$. The TDI bound was about 1.8514% of the estimated mean of T_U_. Thus, 90% of the differences in measurements fell within ±0.67 °C. These numbers suggest no reasonable general agreement between the two methods.

#### Cosinuss One (T_C_) vs. Dräger^®^ TCore (T_B_) temperature monitoring

From the linear mixed model analysis, we got estimated means 36.16 °C (95% confidence interval [35.84, 36.48]) and 36.38 °C (95% confidence interval [36.24, 36.52]) for T_B_ and T_C_, respectively. The variance of T_B_ was 0.4507 and the variance of T_C_ was 0.4338. Their differences had mean 0.2161 °C and standard deviation 0.2713, thus 95% LoA were [-0.32, 0.75]. For evaluation of agreement, the estimates and 95% confidence bounds were, respectively, 0.8708 and 0.7680 for $CCC$, and 0.5668 and 0.7278 for ${TDI}_{0.9}$. The TDI bound was about 2.0126% of the estimated mean of T_B_. Thus, 90% of the differences in measurements fell within ± 0.73 °C. These results suggest no reasonable general agreement between the methods.

#### Cosinuss One (H_C_) vs. ECG (H_D_) heart rate monitoring

The estimated means were 60.18 BPM (95% confidence interval [56.56, 63. 08]) and 60.59 BPM (95% confidence interval [60.40, 60.77]) for H_D_ and H_C_, respectively. The variance of H_D_ was 78.86 and the variance of H_C_ was 75.73. Their differences had mean 0.41 BPM and standard deviation 2.61, thus 95% LoA were [-4.71, 5.53]. For evaluation of agreement, the estimates and 95% confidence bounds were, respectively, 0.9549 and 0.9381 for the $CCC$, and 4.3476 and 4.4828 for ${TDI}_{0.9}$. The TDI bound was about 7.4490% of the estimated mean of H_D_. Thus, 90% of the differences in measurements fell within 4.48 BPM. These numbers suggest no reasonable general agreement between the two methods.

# Additional plots

## Temperature

Figure 1 Trellis plot of temperature data. Points are jittered in x and y direction.

Figure 2 Interaction plot of temperature data.

The trellis plot in Figure 1 leads us to a number of observations. First, Dräger Monitor TempB yields lower readings than Dräger Monitor Temp A and Cosinuss Sensor for most IDs. Second, the differences between the methods vary considerably from ID to ID, suggesting a strong ID $\times$ method interaction. This is confirmed by the interaction plot in Figure 2. Third, the within-subject variation for Dräger Monitor TempB and Cosinuss Sensor readings are similar and higher than for Dräger Monitor TempA. All are small compared to the between-subject variation. Fourth, there is some evidence of nonconstant withing-subject variation over the measurement range. Finally, there might be some outlier measurements in IDs 5 and 7.

## Heart rate

Figure 3 Trellis plot of heart rate data. Points are jittered in x and y direction.

Figure 4 Interaction plot of heart rate data.

The trellis plot in Figure 3 leads us to a number of observations. First, Dräger Monitor and Cosinuss Sensor readings are very similar for most IDs. Second, the differences between the methods seem very constant from ID to ID, suggesting no strong ID $\times$ method interaction. However, the interaction plot in Figure 4 suggests some modest ID $\times$ method interaction. Third, the within-subject variation for Dräger Monitor and Cosinuss Sensor readings are very similar. All are small compared to the between-subject variation. Fourth, there is some evidence of nonconstant withing-subject variation over the measurement range. Finally, there might be some outlier measurements in ID 1.

# Diagnostic plots

## In-ear Sensor ($\boldsymbol{T}_{\boldsymbol{C}}$) vs. Dräger Monitor bladder ($\boldsymbol{T}_{\boldsymbol{U}}$) temperature

Figure 5 Model diagnostics of Bland-Altman analysis of in-ear sensor vs. Dräger Monitor bladder temperature.

## In-ear Sensor ($\boldsymbol{T}_{\boldsymbol{C}}$) vs. Dräger Monitor brain ($\boldsymbol{T}_{\boldsymbol{B}}$) temperature

Figure 6 Model diagnostics of Bland-Altman analysis of in-ear sensor vs. Dräger Monitor brain temperature.

## In-ear Sensor ($\boldsymbol{H}_{\boldsymbol{C}}$) vs. Dräger Monitor heart rate ($\boldsymbol{H}_{\boldsymbol{D}}$)

Figure 7 Model diagnostics of Bland-Altman analysis of in-ear sensor vs. Dräger Monitor heart rate.

# References

Bland JM, Altman DG (2007) Agreement between methods of measurement with multiple observations per individual. J Biopharm Stat 17:571-582

Carstensen B (2010) Comparing Clinical Measurement Methods, vol 59. John Wiley & Sons, Ltd., Chichester, UK. <https://doi.org/10.1002/9780470683019>

Choudhary, Pankaj K, and Haikady N Nagaraja. 2017. *Measuring Agreement*. Wiley Series in Probability and Statistics. Hoboken, NJ, USA: John Wiley & Sons, Inc. <https://doi.org/10.1002/9781118553282>.

Olofsen, Erik, Albert Dahan, Gerard Borsboom, and Gordon Drummond. 2015. “Improvements in the application and reporting of advanced Bland–Altman methods of comparison.” *J Clin Monit Comput* 29 (1): 127–39. <https://doi.org/10.1007/s10877-014-9577-3>.

Thomas, James D., and Robert A. Hultquist. 1978. “Interval Estimation for the Unbalanced Case of the One-Way Random Effects Model.” *Ann Stat* 6 (3): 582–87. <https://doi.org/10.1214/aos/1176344202>.

Zou, GY. 2013. “Confidence interval estimation for the Bland–Altman limits of agreement with multiple observations per individual.” *Stat Methods Med Res* 22 (6): 630–42. <https://doi.org/10.1177/0962280211402548>.
